# Report of Amputation of the Thigh, Etc.

**Published:** 1873-08

**Authors:** De Saussure Ford

**Affiliations:** Professor of Anatomy in the Medica College of Georgia


### REPORT OF AMPUTATION OF THE THIGH AT THE MIDDLE THIRD, FOR ENCEPHALOID TUMOR.

BY D'SAUSSURE FORD, M.D.,
*Professor of Anatomy in the Medica College of Georgia.*

[From the Transactions of the Medical Association of Georgia, 1873.]

*Mr. President*,—The classification of tumors is one of interest, generally, but particularly, since it is yet veiled in some obscurity, and this may arise from the yet unacknowledged claims of cellular pathologists, by those of different, but equally commendable, pathological schools. When such men as Virchow and Paget, scientists of eminence, whose lives are devoted to inquiry, and whose researches have thrown such meteoric light upon heretofore perplexing questions in pathology—seeking for, and enjoying the privileges of abundant material for investigation — acknowledge the difficulties in differential diagnosis of tumors, it is not surprising that younger men who only occasionally meet with, and yet are compelled to treat these morbid growths, hesitate, and are often bewildered, in diagnosis, treatment, and prognosis.

I present this specimen of tumor to the society, believing it to be a malignant one of the encephaloid variety—a part of it is osseous, a part the true medullary cancer—and it is of interest, as showing that the heads of the tibia and fibula are, in a diseased condition, very much enlarged. This is, however, not unusual. Indeed, pathologists intimate clearly that such a state is by no means rare, rather the general rule, that this variety of tumors usually take origin in the bone; the periosteum and connective tissues are then contaminated, and the rapid, or even slow, proliferation of cells goes on, those forming these usually extensive growths reaching the enormous dimensions of thirty-six inches in circumference, the size of one which I have recently had the opportunity of examining. On the other hand, Paget believes that "the disease begins simultaneously in all these parts, or, at least, when they are affected in succession, it is not generally by extension from one to the other." Whether the one or the other doctrine be correct, I will not detain this convocation by discussing, but very evidently in this case, the history of which will be detailed hereafter, the disease can be explained according to the doctrine of Paget.

It will probably be admitted that any attempt to extirpate this mass would have failed, and if the patient had survived the dissection, the disease would have been renewed with violence; but it is a question of importance to speculate, and possibly determine what may be the ultimate issue to the patient, after the amputation of the limb, which, as you perceive, was the treatment adopted.

Amputation for the relief of the subject of malignant tumor is no novel proceeding, and yet the disease may reappear in the stump, or, if not there, after an indefinite period, attack more central and vital organs. The celebrated case of Dr. Mussey illustrates both these sequelæ. The arm was amputated for encephaloid, involving the hard and soft tissues of the elbow; the disease returning and implicating the head of humerus, glenoid cavity, and scapula, extremity of the clavicle, of the same side, a subsequent operation was performed upon the remaining portion of the humerus, head of scapula and extremity of the clavicle, when the disease did not return near these parts, but did finally involve the lungs.

Fifteen years ago I amputated the thigh for a large osteo-sarcoma, the tibia, fibula, and condyles of the femur diseased, and five years ago the man was in perfect health. If it had returned in the stump, I should have as heroically pursued the disease as did Dr. Mussey.

The causes of this disease, and other kindred malignant affections, are often obscure. They may be hereditary, and hence the importance of examining, with annoying scrutiny it may be, into the family record, for if hereditary, the prognosis may not be favorable, and rather lead to the idea of its constitutional rather than direct local origin. Mr. Erichsen says these encephaloid tumors "occur without any exciting cause, when it is, evidently, as in hereditary and connate cases, the result of some constitutional condition." "In many cases, however, *it can be distinctly traced to some exciting cause, being immediately occasioned by a blow, injury, or other violence.*" "It is a question whether external causes can give rise to cancer, without the previous existence of constitutional predisposition to disease." After discussing this question with his usnal ability, the distinguished surgeon believes "that cancer is primarily local, and only becomes secondarily constitutional by absorption into the system."

"This opinion," he continues. "Velpeau strongly supports, and it is founded on several reasons," and of these he remarks: "These arguments appear to me to be conclusive as to cancer being, primarily, a local disease, and only becoming constitutional, secondarily, by contamination of the blood, and absorption into the system, and, consequently, to justify operation for the removal of cancerous tumors in suitable cases."—*Vide Erichsen's Surgery, last edition, page* 495.

Whether or not this doctrine of "contamination of the blood" from "local disease" as the cause of "constitutional" disturbance, will agree with Virchow's cellular theory, I will not here discuss, but refer to the writings of the latter pathologist.

Our case will illustrate, I think, the fact that the disease was local, since the irritation caused by the final blow, to an already weak point, was sufficient to invite an energetic proliferation in those cellular elements about the region, for that they do exist there I believe, with Virchow, who declares that there is "scarcely any parts of the body which do not possess cellular elements."

Case.—Mr. G——, of Elbert county, Georgia, aged forty-five, presented himself for treatment, February 10th, 1873, having a large elastic tumor occupying the superior thirds of the tibia and fibula, the mass presenting backward and upward, toward the popliteal space.

Twenty years ago, after diving from a flat-boat, immediately he experienced pain behind the knee-joint, which continued to annoy him now and then, for fifteen years. There was slight immobility of the knee-joint, but not perceptible enlargement, and not interrupting the active duties of the farmer, such as ploughing, etc.

Two years ago, in attempting to drive depredating cattle from a corn-patch, he was thrown violently down, by a rail tripping him, in some way, and "flying up," it struck him upon the back of the knee.

From this time he suffered much, and soon was discovered an enlargement, which had developed to the dimensions you now see.

The patient had been using large doses of morphine for more than a year, to quiet the agony, and this had so disturbed digestion that he was much emaciated. The tumor was weighty; his nervous energies were almost paralyzed; his life a very burden. The most searching inquiry elicited no family taint, so, after consulting with my distinguished associate, Professor L. A. Dugas, it was decided to amputate the limb, at the middle of the middle third of the femur.

Sulphuric ether was administered to compel anæsthesia, and I operated by the circular method, before the students of the Medical College of Georgia, February 12th, 1873. The stump did well, and on the fifth week he was discharged from the hospital. Since that time I have heard he was continuing to do well.

Whether or not the disease will return in the stump, or attack either the lungs, or liver, I can not positively declare, but believe that the man's life has been prolonged by such a surgical procedure, and if so, the thought will ever be a gratification, independent of any cavils from those surgeons who would have hesitated to interfere.

